# Seroprevalence and Risk Factors Associated with *Toxoplasma gondii* Infection in the Population Referred to Rural and Urban Health Care Centers in Zahedan, Primary Referral Level, in Southeastern Iran

**DOI:** 10.1155/2022/7311905

**Published:** 2022-05-12

**Authors:** Samaneh Abdolahi Khabisi, Seideh Zeinab Almasi, Siavash Liravi Zadeh

**Affiliations:** ^1^Dept. of Parasitology and Mycology, Faculty of Medicine, Zahedan University of Medical Sciences, Zahedan, Iran; ^2^Infectious Diseases and Tropical Medicine Research Center, Research Institute of Cellular and Molecular Sciences in Infectious Diseases, Zahedan University of Medical Sciences, Zahedan, Iran; ^3^Department of Epidemiology and Biostatistics, School of Public Health, Zahedan University of Medical Sciences, Zahedan, Iran

## Abstract

**Introduction:**

Toxoplasmosis is one of the protozoan diseases caused by *Toxoplasma gondii*. This study is aimed at evaluating the seroprevalence and associated risk factors of *Toxoplasma gondii* infection in the population referred to rural and urban health care centers in Zahedan, southeast Iran.

**Methods:**

A total of 1,324 blood samples of patients referred to the health care centers were evaluated using the IgG Toxoplasma ELISA Kit, between October 2019 and August 2021. The obtained data were analyzed through univariable and multivariable regression models.

**Results:**

The seropositivity of *Toxoplasma gondii* infection was obtained at 18.8%. In the multivariable logistic regression model, risk factors including age group of 11-30 (OR = 3.25, 95% CI: 1.29-7.06), urban residency (OR = 4.36, 95% CI: 2.9-6.3), students (OR = 3.76, 95% CI: 1.88-4.53), and contact with cat (OR = 7.67, 95% CI: 4.76-12.36) were significantly associated with seropositivity to *Toxoplasma gondii* infection. Moreover, consumption of washed vegetables with salt or detergents decreases (OR = 0.14, 95% CI: 0.09-0.23) the risk of *Toxoplasma gondii* infection. According to the results of the multivariable logistic regression, no significant association was observed between seropositivity to *Toxoplasma gondii* and other risk factors.

**Conclusion:**

The results of this study indicated significant seropositivity to *Toxoplasma gondii* infection in the population referred to rural and urban health care centers in Zahedan, Iran. Therefore, health programs should be considered for raising awareness regarding the risk factors for *Toxoplasma gondii* infection in this region.

## 1. Introduction

Toxoplasmosis is caused by an intracellular protozoan (*Toxoplasma gondii*) and can infect humans and many warm blood animals. In developed and developing countries, the seroprevalence of this disease is estimated at 30%-50% in the human population [1, 2]. Geographical conditions of the region, dietary habits, and the level of host immunity play prominent roles in the seroprevalence of human toxoplasmosis. Toxoplasmosis is asymptomatic and sometimes mild in individuals with a competent immune system; however, in patients with immune deficiency, it can have severe symptoms, including encephalitis, chorioretinitis, and myocarditis [3, 4]. In the recently conducted studies, latent toxoplasmosis has been identified as an important and effective factor in the development of diseases, such as Parkinson's, Alzheimer's, bipolar, and schizophrenia [5, 6]. Therefore, the screening for anti-toxoplasma antibodies in different groups is useful to control and prevent the complications of this disease. So far, most studies on the prevalence of toxoplasmosis in Iran have been performed on high-risk groups, including immunocompromised individuals and pregnant women [7, 8].

In the southeast of Iran, Sistan and Baluchistan province, Zahedan city, several studies have been conducted on seroprevalence of toxoplasmosis in the population of blood donors [9], pregnant mothers [10], hemodialysis patients [11], and patients undergoing chemotherapy for malignancies [12]. The seroprevalence of toxoplasmosis varies in healthy individuals in different provinces of Iran. One of the most rational reasons for designing this study is the lack of sufficient information about the recent seroprevalence to *Toxoplasma gondii* infection in healthy individuals of the rural and urban population in Zahedan. Moreover, this study is aimed at evaluating the human seroprevalence to *Toxoplasma gondii* infection and its relative epidemiological risk factors in the population referred to rural and urban health care centers in Zahedan, southeast Iran.

## 2. Materials and Methods

### 2.1. Study Design and Participants

This cross-sectional study was conducted between October 2019 and August 2021 after getting approval code, https://ethics.research.ac.ir/IR.ZAUMS.REC.1401.036, from the Ethics Committee of Zahedan University of Medical Sciences, Zahedan, Iran. Participants were people referred to urban and rural health care centers. Health care centers provide the first level of care for the urban and rural population in Iran. The major responsibilities delegated to these centers include vaccination, midwifery practices, family planning, newborn screening, screening for hypertension and diabetes, environmental health, occupational health, disease control, school health, oral–dental health, monitoring occupational health standards, case finding, first aids, follow-up, and urgent referrals.

According to the statistical information of the Vice Chancellor for Health of Zahedan University of Medical Sciences, about 45 to 50% of the urban and rural population of Zahedan refer to health care centers annually and receive these services each year. After obtaining the consent of the participants and filling out a questionnaire, 2 ml of peripheral blood samples was collected from each participant. A checklist of demographic characteristics and related risk factors for *Toxoplasma gondii* infection, including gender, age, job, contact with cat, contact with soil, consumption of raw meat, and vegetable washing method, was prepared and each participant was requested to fill out the form. Pregnant mothers and patients with malignancy, HIV/AIDS, and other immunodeficient disorders were excluded from the study.

The sample size was calculated based on *Toxoplasma gondii* seroprevalence rate of 23% [9], confidence level of 95%, *α* = 0.05 and marginal error of 0.03%, and formula of *N* : *z*^2^_1−*α*/2_ *p*(1 − *P*)/*d*^2^. A total of 1,324 blood samples were collected from 509 and 815 rural and urban communities, respectively. Zahedan has 44 urban health care centers, including 12, 14, 8, 4, and 6 in the northeast, northwest, southwest, southeast, and center, respectively. Furthermore, there are 10 rural health care centers in Zahedan. We used a simple randomized cluster method for sampling. The number of clusters from rural or urban health care centers was calculated using the statistical formula number of health centers in each area/total number of health centers × number of health centers in each area. A total of 815 blood samples were collected from four, five, one, three, and two northeast, northwest, southwest, southeast, and center urban clusters, including 269, 281, 56, 98, and 111 samples, respectively. In addition, from seven rural health care centers including Sarjangal, Shuro, Allahabad Doomak, Garage, Haji Abad Cheshmeh, Roshanabad, and Chah Zard, a total of 509 blood samples were collected, including 101, 31, 116, 50, 100, 46, and 65 samples, respectively. The required number of samples in each center was determined according to their household size. Collected blood samples were sent to the parasitology laboratory of Zahedan Medical University based on cold chain (4-8°C) conditions.

### 2.2. Serological Analysis

Blood samples were centrifuged at 1,000 *g* for 3 min. Subsequently, the sera were stored at -20°C until serological testing. All samples were evaluated for anti-toxoplasma IgG antibodies with an indirect ELISA kit (Pishtaz Teb Diagnostics, Tehran, Iran). The sensitivity and specificity of this kit are over 95%.

### 2.3. Statistical Analysis

The obtained data were analyzed in SPSS software (version 22). A univariable logistic regression model was performed to find out the association between the potential risk factors and seropositivity to *Toxoplasma gondii* infection. Odds ratios (OR) and 95% confidence intervals (CI) were used to calculate the association among the potential risk factors. Variables with *P* value < 0.20 in univariable analysis at 95% CI were analyzed using the multivariable logistic regression model. Goodness of fit for logistic regression model was analyzed using the Hosmer–Lemeshow test. The VIF (Variance Inflation Factor) index was reported for collinearity between variables. Also, the confidence interval of seroprevalence of *Toxoplasma gondii* infection was calculated using Stata software.

## 3. Results

The VIF index showed no collinearity between variables (Table 1). Also, the Hosmer–Lemeshow test showed no significant difference between the observed and expected values (chi‐square = 5.40, *P* value = 0.71).

The majority of the participants (*n* = 273; 67.3%) were female. Blood samples were collected from a total of 815 (61.6%) and 509 (38.4%) people referred to urban and rural health centers in Zahedan, Iran, respectively. In this study, 250 out of 1,324 samples had IgG antibodies against *Toxoplasma gondii*. The seropositivity to *Toxoplasma gondii* infection was obtained at 18.8% (95% CI: 16.86%-21.08%). Among seropositive cases, 67.2% and 32.8% of patients were residents in urban and rural areas in Zahedan, respectively. The results of the univariable logistic regression test showed that the age group 11-30 (OR = 2.93, 95% CI: 1.35-6.35) years had a high risk for *Toxoplasma gondii* infection. Moreover, urban residents had a high risk (OR = 1.35, 95% CI: 1.01-1.80) for *Toxoplasma gondii* infection, compared to the rural residents. The results also revealed that contact with a cat significantly increased (OR = 2.84, 95% CI: 2.14-3.76) the risk of *Toxoplasma gondii* infection. Among job groups, students are more (OR = 2.69, 95% CI: 1.45-4.88) at risk for *Toxoplasma gondii* infection. In the multivariable logistic regression test, the age group of 11-30 (OR = 3.25, 95% CI: 1.29-7.06) years had a high risk for *Toxoplasma gondii* infection. Regarding the job status, students were 3.76 times more likely to be infected with *Toxoplasma gondii* than farmers (OR = 3.76, 95% Cl: 1.88-4.53).

According to the results of the multivariable logistic regression, no significant association was observed between seropositivity to *Toxoplasma gondii* infection and gender (*P* = 0.44). The results revealed that contact with cats significantly increases (OR = 7.67, 95% CI: 4.76-12.36) the risk of *Toxoplasma gondii* infection. Also, meat consumption (OR = 1.44, 95% CI: 0.88-2.30) and contact with soil (OR = 1.67, 95% CI: 0.96-2.90) increase the chances of contracting *Toxoplasma gondii* infection. However, there is insufficient evidence to support the hypothesis of a relationship between meat consumption and the chance of getting *Toxoplasma gondii* infection.

Table 1 shows the demographic characteristics and relative risk factors to *Toxoplasma gondii* infection analyzed using univariable and multivariable logistic regression models.

## 4. Discussion

In the present cross-sectional study, the human seropositivity to *Toxoplasma gondii* infection in the population referred to rural (6.2%) and urban (12.6%) health care centers was reported at 18.8%. The seropositivity to *Toxoplasma gondii* infection in this region is lower than that in the general population (39%) in Iran [13, 14]. The seropositivity of the present study is consistent with that in the previous studies conducted on 375 blood donors (25%) and 119 healthy individuals (23%) in Zahedan, Iran [9, 11]. The seropositivity to *Toxoplasma gondii* infection in this study is lower than that in 221 pregnant women (30.8%), 119 hemodialysis patients (44.5%), and 154 patients undergoing chemotherapy (39.4%) in Zahedan, southeast Iran [10–12]. Furthermore, the seroprevalence obtained from the present study is lower than that reported from Iran's neighbor countries, including Qatar (29.8%), Pakistan (29.48%), and Turkey (31.9%) [15–17].

Zahedan has dry and hot summers, as well as dry and cold winters. In Iran, especially in the southeast areas, severe drought has been also experienced in recent years. This weather condition in southeastern Iran is an essential factor for the low seroprevalence of *Toxoplasma gondii* infection compared to other regions. Oocyst sporulation and survival require optimal conditions, such as adequate heat and humidity; however, these factors are not conductive in this area.

In the present study, risk factors, including the age group of 11-30 years, place of residency, contact with cats, and job status showed a significant relationship with *Toxoplasma gondii* infection in both univariable and multivariable logistic regression models. The age group of 11-30 years had 3.25 times more chance to become infected with *Toxoplasma gondii* than younger age groups. In this respect, the results of this study are consistent with the findings of the previous studies conducted in the west and north of Iran and other countries [18–22].

In our study, seropositivity to *Toxoplasma gondii* infection was significantly associated with the job, and the most seroprevalence to *Toxoplasma gondii* infection was reported in students, employees, and housekeepers. Playgrounds of students in Zahedan are mostly dirt and are likely to be infected with *Toxoplasma gondii* sporulated oocytes. Additionally, housekeepers are more at risk to become infected with *Toxoplasma gondii* because of keeping pets, such as cats, and cleaning contaminated vegetables. In this regard, the results of this study are in line with the findings of other studies in Iran and other countries [14, 19, 23, 24].

In this study, among all of the risk factors, washing vegetables with salt and detergents significantly decreased the chance of getting *Toxoplasma gondii* infection. In this respect, this result is consistent with the findings of other studies [19, 25, 26]. The consumption of unwashed or inappropriately washed vegetables plays a vital role in transmitting *Toxoplasma gondii* oocysts. In this respect, this result is in line with the studies reported from north of Iran, Mexico, and Ethiopia [19, 20, 27].

In this study, residency in urban areas showed a significant association with seropositivity to *Toxoplasma gondii* infection. Urban residents of Zahedan are more exposed to the sources of *Toxoplasma gondii* infection. Although the culture of Iranians living in big cities has changed in recent years and cats have entered the houses, the religious beliefs of the rural residents of this region have prevented them from accepting this cultural change and having close contact with cats and dogs.

Close contact with cats is the main factor for *Toxoplasma gondii* infection in humans. In this study, a significant association was reported between contact with cats and seropositivity to *Toxoplasma gondii* infection. In this regard, the results of the present study are in line with the findings of other studies [19, 20, 27].

Raw meat consumption is one of the sources of human infection to toxoplasmosis. In this study, no significant relationship was reported between seropositivity to *Toxoplasma gondii* infection and raw meat consumption. In the study area, local dishes, such as Champ, Tanoorcheh, and Beryani, are used with undercooked meat. However, due to economic poverty, most people in this area are unable to eat meat, as the predominant food. In this respect, the results of some studies are consistent with the findings in our study [20, 28]. In contrast to our study, in previous studies conducted in the north of Iran, Mexico [22], and Slovakia [29], there was a significant association between seropositivity to toxoplasmosis and raw meat consumption.

Among the notable limitations of this study, we can refer to the selection of the population. The sampling from people living in rural houses was not possible, because of the financial constraints of the project, the long distance of rural houses from the city, the impassability of roads, and epidemic conditions of COVID-19. Therefore, the results of this study can only be generalized to the people referred to rural and urban health care centers in Zahedan. The lack of sufficient information about the type of meat consumed by participants should be considered another limitation of this study.

Regarding the limitations in this study, due to financial restrictions, IgM antibodies against *Toxoplasma gondii* were not evaluated. Furthermore, the IgG antibodies against *Toxoplasma gondii* remain positive for many years, and lifestyle and other risk factors may be changed over time. Therefore, analysis of the risk factors based on IgG antibodies against *Toxoplasma gondii* cannot establish a direct relationship between behaviors and toxoplasmosis.

In conclusion, our results showed the low seropositivity to *Toxoplasma gondii* infection in the population referred to rural and urban health care centers in Zahedan, southeast Iran. Risk factors, including age, consumption of unwashed vegetables, job, and contact with a cat, increase the chance of *Toxoplasma gondii* infection. Therefore, health programs to consider the risk factors for *Toxoplasma gondii* infection should be given more attention.

## Figures and Tables

**Table 1:** Seroprevalence of *Toxoplasma gondii* infection according to sociodemographic characteristics estimated by univariable and multivariable logistic regression models.

Variables		No. of participants (%)	No. of seropositives (%)	Univariable		Multivariable		Collinearity statistic
				OR_unadjusted_ (95% CI)	*P* value	OR_adjusted_ (95% CI)	*P* value	VIF
Age	≤10	51 (4.7)	8 (3.2)	1 (ref)		Ref (1)		1.08
	11-30	346 (32.2)	173 (69.2)	2.93 (1.35-6.35)	0.006	3.25 (1.29-7.06)	0.01	
	31-50	397 (37)	55 (22)	0.84 (0.36-1.8)	0.61	0.68 (0.28-1.69)	0.00	
	≥51	280 (26)	14 (5.6)	0.29 (0.11-0.72)	0.008	0.16 (0.6-0.48)	0.00	
Sex	Male	351 (32.7)	72 (28.8)	1 (ref)		1 (ref)		1.30
	Female	723 (67.3)	178 (71.2)	0.83 (0.61-1.12)	0.23	1.92 (0.75-1.87)	0.44	
Job	Farmer	133 (12.4)	18 (7.2)	1 (ref)		Ref (1)		1.30
	Other	257 (24)	52 (20. 8)	1.49 (0.84-2.65)	0.17	2.43 (1.15-5.16)	0.02	
	Rancher	117 (10.9)	27 (10.8)	1.70 (0.89-3.25)	0.10	2.67 (1.24-5.73)	0.01	
	Housekeeper	328 (30.5)	79 (31.6)	1.78 (1.02-3.08)	0.04	2.76 (1.44-5.28)	0.00	
	Employee	110 (10.2)	27 (10.8)	1.81 (0.94-3.44)	0.07	2.06 (1.06-4.02)	0.03	
	Student	129 (12)	47 (18.8)	2.69 (1.45-4.88)	0.01	3.76 (1.88-4.53)	0.01	
Residence	Rural	427 (39.7)	82 (32.8)	1 (ref)		1 (ref)		1.41
	Urban	647 (60.3)	168 (67.2)	1.35 (1.01-1.80)	0.04	4.36 (2.9-6.3)	0.000	
Vegetable washing method	Water	421 (39)	50 (20)	1 (ref)		1 (ref)		1.33
	Salt and detergent	653 (61)	200 (80)	2.57 (1.84-3.59)	0.001	0.14 (0.09-0.23)	0.000	
Raw meat consumption	No	542 (50.5)	115 (46)	1 (ref)		Ref (1)		1.08
	Yes	532 (49.5)	135 (54)	1.96 (0.90-1.57)	0.2	1.44 (0.88-2.3)	0.14	
Contact with cat	No	774 (72)	119 (47.6)	1 (ref)		1 (ref)		1.04
	Yes	300 (28)	131 (52.4)	2.84 (2.14-3.76)	0.000	7.67 (4.76-12.36)	0.000	
Contact with soil	No	126 (11.7)	21 (8.4)	1 (ref)	0.12	1 (ref)		1.08
	Yes	948 (88.3)	229 (91.6)	1.44 (0.89-2.35)		1.67 (0.96-2.9)	0.06	

## Data Availability

All data used to support the findings of this study are available from the corresponding author upon request.
